# Atrazine Degradation by the Nano-Shielded Carbon Dots–Lacassa (CDs-S-M-Lac) System

**DOI:** 10.3390/nano16150943

**Published:** 2026-07-30

**Authors:** Carlos A. Guerrero-Fajardo, David Bocanegra-Cardenas

**Affiliations:** 1 Departamento de Química, Facultad de Ciencias, Universidad Nacional de Colombia, Cra 30 No. 45-03, Bogotá 111321, Colombia; dabocanegraca@unal.edu.co; 2 Departamento de Ingeniería, Facultad de Ingeniería, Universidad Nacional de Colombia, Cra 30 No. 45-03, Bogotá 111321, Colombia

**Keywords:** carbon dots, lacassa, atrazine, degradation, sustainable development goals, sustainability

## Abstract

Atrazine is one of the most toxic herbicide pollutants for ecosystems and the environment, as its persistence poses a risk to environmental health. Therefore, this research focuses on the production of a series of shielded nanostructured compounds known as carbon dots. To this end, three different shielded nanomaterials will be synthesized, namely carbon dots (CDs-S), magnetized carbon dots (CDs-S-M), and laccase-catalyzed magnetized carbon dots (CDs-S-M-Lac), the latter being the biocomposite. During the characterization of these materials, stretching vibrations corresponding to the hydroxyl group were obtained in the 3100–3600 cm^−1^ region, as well as 1750 cm^−1^ bands associated with oxygenated carbonyl and carboxylate groups, which are important in the adsorption processes of contaminants on the material’s surface. Biocatalytic activity tests were also performed on a total volume of 3.0 mL, contained in an atrazine solution with an initial concentration of 10 mg·L^−1^. Raman analysis revealed bands in the D (1350 cm^−1^) and G (1580 cm^−1^) regions, characteristic of ${SP}^{2}$ hybridizations associated with carbonaceous materials, thus describing a material capable of degrading contaminants with a capacity of approximately 14 mg/L; this degradation is achieved with CDs-S-M-Lac.

## 1. Introduction

**Figure 1:**
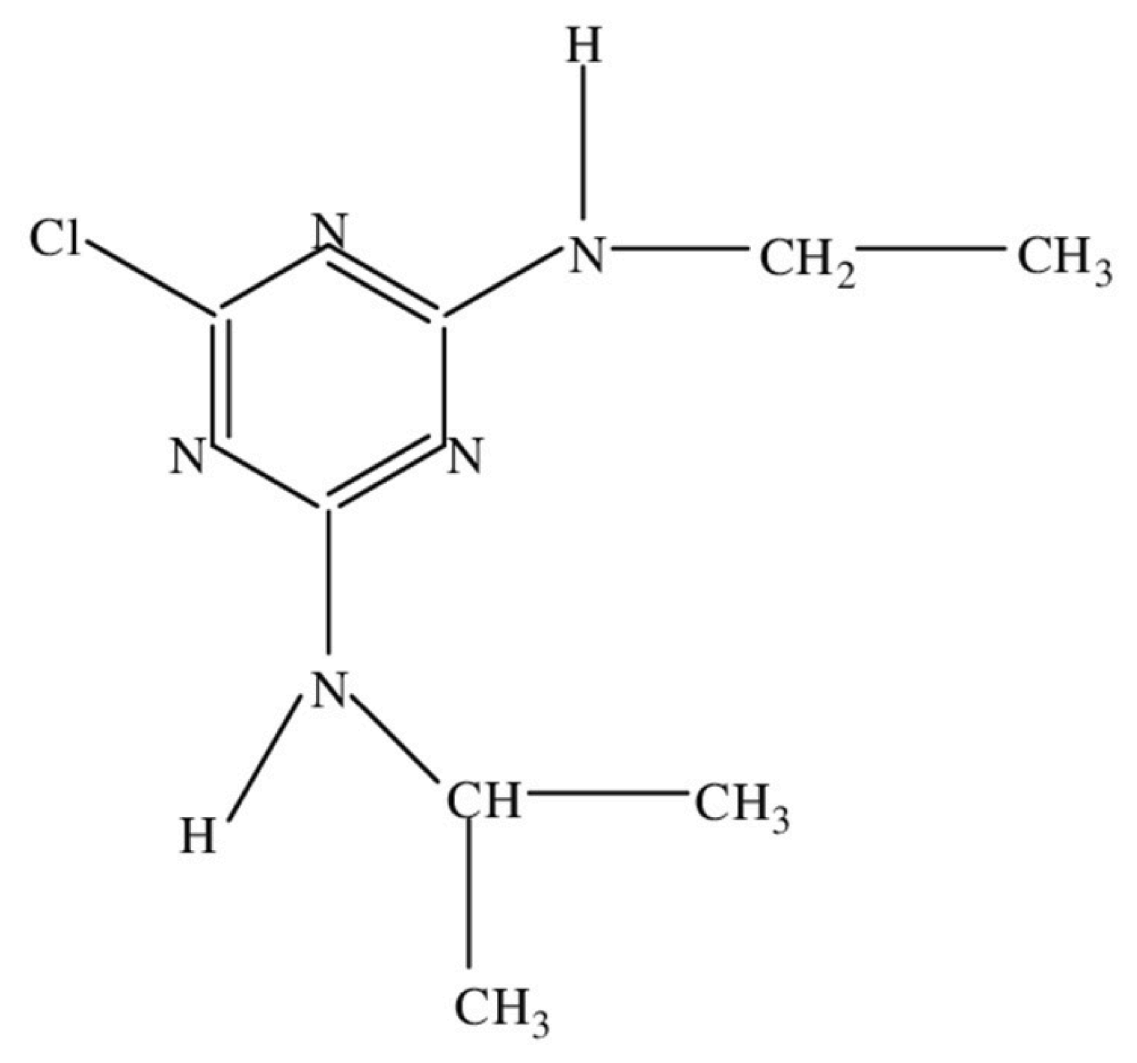
Atrazine structure.

Atrazine (2-chloro-4-ethylamino-6-isopropylamino-1,3,5-triazine; C_8_H_14_ClN_5_) is a s-triazine herbicide, generally used to regulate broadleaf as well as grassy weeds (Figure 1). Atrazine is one of the most harmful chemical pollutants for the environment and its ecosystems, currently classified as one of the most toxic herbicides in the agricultural sector, with an annual global use of 70.000–90.000 tons. Atrazine is currently one of the two most widely used pesticides in the United States (Florida being an example), especially for weed control in corn and sorghum, although it is also used on vegetables, grains, fruits, and nuts. It is the most extensively studied herbicide as a toxic contaminant in freshwater sources [1,2]. This contaminant can be transmitted through food or drinking water, although these are not a significant source of such contaminants [2]. This compound is characterized by having no odor; it is slightly alkaline and non-flammable. Structurally, its nitrogen atoms in the aromatic ring of atrazine make it toxic and persistent in the environment. Although the United States uses it extensively, it is not the only country that uses this herbicide extensively, as Brazil and China are widely known for its use in agriculture [3].

Atrazine is commonly used to control the growth of annual grasses and broadleaf weeds. This herbicide inhibits photosynthesis in plants by preventing electron transfer at site II of the chloroplast complex, exhibiting low toxicity to non-photosynthetic organisms such as heterotrophs [4]. Atrazine is one of the most widely used pollutants in the world and, consequently, persists in water and soil. Therefore, the need to develop safe and efficient disposal strategies is paramount, given their importance to ecosystems and public health. A clear example of high atrazine contamination in agricultural soils is in China, where concentrations of this compound have exceeded the permitted limit (1.0 mg/kg), reaching levels of 1.86 to 1100 mg/kg [5].

**Figure 2:**
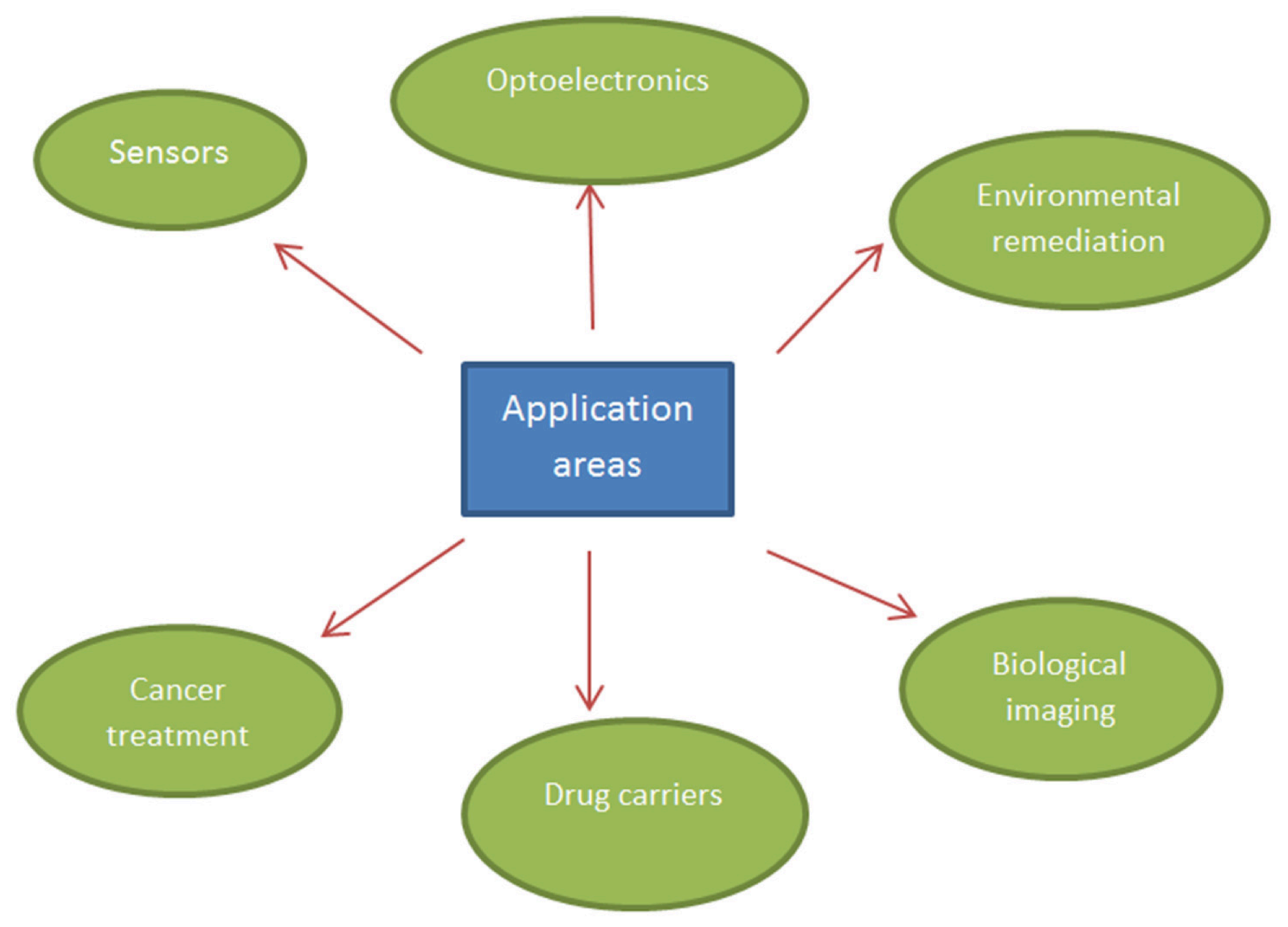
Applications of carbon dots (CDs) [7].

Therefore, it is important to develop innovative technologies that allow for the degradation, adsorption, and/or degradation of atrazine from water sources. This research aims to develop carbon dot compounds (also known as carbon quantum dots), a class of carbon nanoparticles smaller than 100 nm. Several subgroups exist within this class based on their crystalline and morphology. Carbon dots (CDs) offer unique physical, chemical, and optical properties in their structure [5,6]. These materials have broad applications, including in health fields such as cancer treatments, sensors, drug production, and environmental remediation [7]. This research will focus on the latter, as it aims to develop different types of carbon dots (CDs), some coupled with laccase and magnetite (Figure 2).

The importance of this research lies in its use of resources that are not currently relevant to the industrial sector, but also in its innovative approach to current research, which is crucial in the world of materials. Furthermore, it contributes to innovation, as new technologies are being discovered that solve environmental problems, and if scaled up further, it could even lead to products that are safe for human health.

## 2. Materials and Methods

The reagents used were of analytical grade and were purchased from Thermo Fisher Chemical or Sigma Aldrich. These included analytical-grade ferric chloride hexahydrate (FeCl_3_ · 6H_2_O), sucrose (C_12_H_22_O_11_), urea (CO(NH_2_)_2_), and polyethylene glycol 3350 (PEG 3350), also known as ${\left(H(OCH_{2}CH_{2}\right)}_{n}$OH; where n = 3350). Mono- and dibasic phosphates (NaH_2_PO_4_·6H_2_O and Na_2_HPO_4_·12H_2_O) and citric acid (C_6_H_8_O_7_) were used to prepare the Sörensen phosphate buffer. The atrazine used was purchased commercially from a local supplier. All the water used in the experiments was double-distilled water. It is important to note that the instruments must be completely sterilized and clean, without any trace of dirt, since carbon dots are extremely sensitive to any solid residue, thus affecting the accuracy of the analyses.

### 2.1. CDs, CDs-M y CDs-S-M-Lac Preparation

Carbon dots (CDs-S) were synthesized using a hydrothermal reaction between sucrose and urea, which is explained underneath of the text. Magnetized carbon dots (CDs-M) were produced similarly, and as a result CDs-S-M-LAC is preparate, which leads to a dark brown suspension due to magnetization. The methodology is described in detail in Table 1 below.

### 2.2. Physicochemical Characterization

✓ **FE-SEM, SEM-Lyra:** Field emission scanning electron microscopy was performed to observe the surface morphology of the CDs-S, CDs-M, and CDs-S-M-Lac materials. This is done to obtain detailed, high-resolution images of carbon dots, as these are critical for these materials (CDs) because they allow for the characterization of their size (generally <10 nm, as is the case here), shape, crystallinity, and surface structure. This direct visualization is essential for optimizing their synthesis, since, as mentioned earlier, the sensitivity of the samples is crucial [8,9].
✓ **UV–VIS Spectroscopy:** Spectrophotometry was recorded using a Thermo Scientific and BioMate 160 spectrophotometer in the spectral range of 190–400 nm. It allows us to confirm its synthesis, determine its purity and analyze its optical structure (electronic transitions such as π-π y n-π), and in the present research it aims to identify surface functional groups, which is important in determining active sites of the carbon points [10].
✓ **FTIR spectroscopy:** Data were obtained using a SHIMADZU^®^ IR Tracer-100 spectrometer, registering wavelengths between 4000 and 400 cm^−1^ at a scan rate of 0.2 (cm·s)^−1^. This is fundamental for characterizing carbon dots (CDs), as it identifies the surface functional groups (–OH, –COOH, –NH_2_, C=O, C–N) that determine their solubility, biocompatibility, and optical properties. It allows for the verification of functionalization and analysis of the chemical structure after synthesis [11,12].
✓ **Raman spectroscopy:** Data were obtained using a Horiba^®^ X-Plora Raman spectrometer coupled to an Olympus BX41 microscope, equipped with three lasers (785 nm, 638 nm, and 532 nm). Raman spectroscopy is crucial for carbon dots (CDs) because it allows for the non-destructive characterization of their crystalline structure, degree of disorder, and surface functionalization. It identifies the D (disorder) and G (graphic) bands, determining the intensity ratio to evaluate the material’s quality and structure [13].

## 3. Results

### 3.1. Summary of the CDs-S, CDs-M and CDs-S-M-Lac

For this, carbon quantum dots were synthesized as an enzymatic nano protection system. Sucrose, urea, and polyethylene glycol were used as carbon sources, while urea and FeCl_3_ · 6H_2_O acted as precursors for magnetite formation.

**Figure 3:**
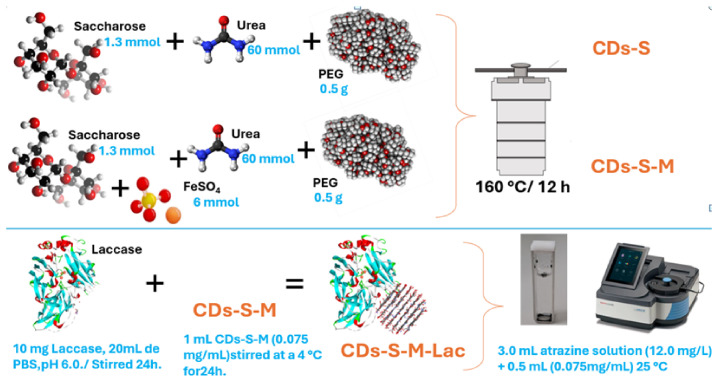
CDs and CDs-S-M synthesis protocol and their application as support for laccase immobilization [14].

In both the CDs-S and CDs-S-M cases, hydrothermal treatment resulted in a dark brown suspension (mentioned previously), indicating the formation of carbonaceous nanomaterials with conjugated domains and surface functional groups (Figure 3).

### 3.2. Physicochemical Characterization of CDs-S, CDs-S-M y CDs-S-M-Lac

**Figure 4:**
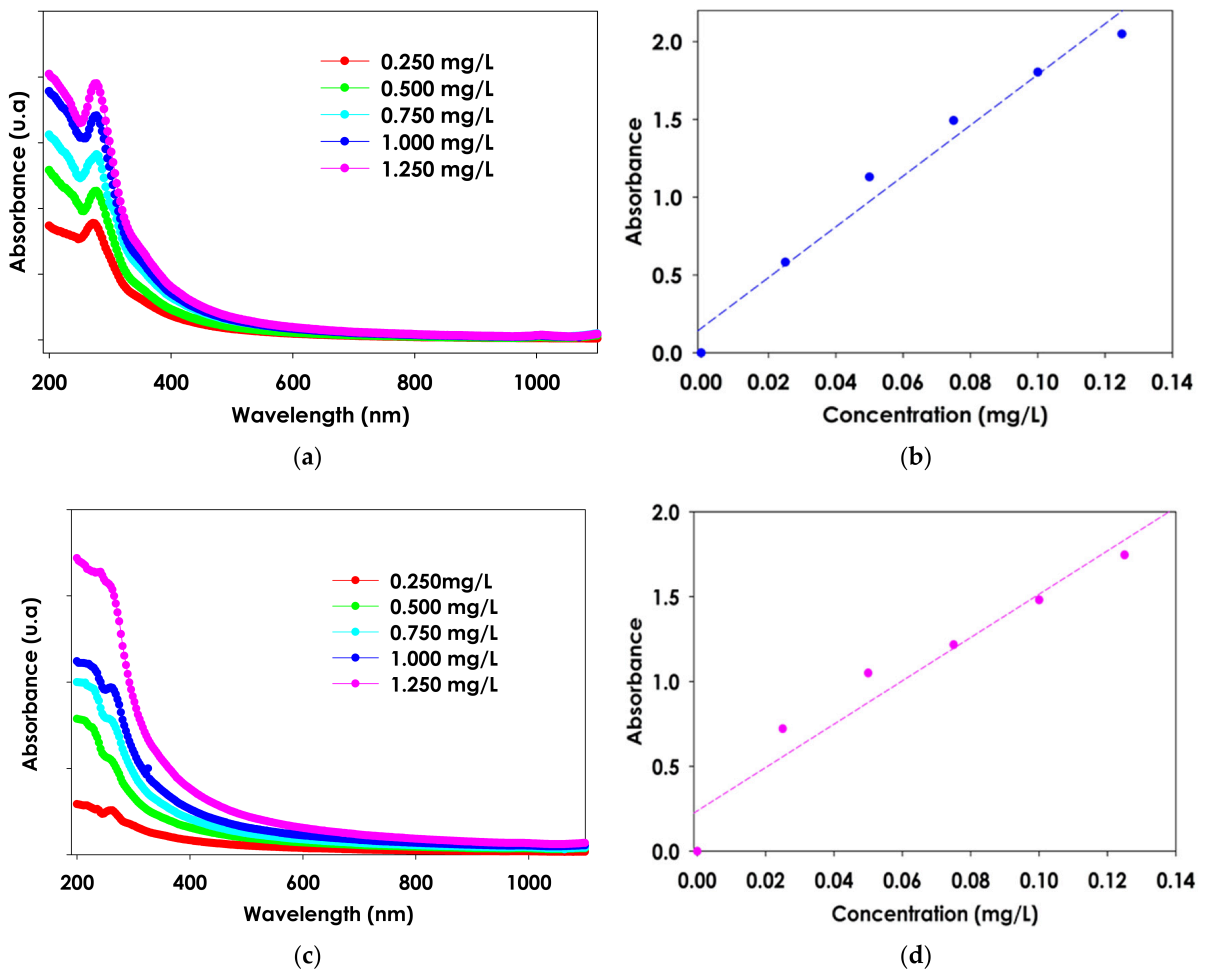
(**a**–**c**) UV–visible absorption spectra of CDs-S and CDs-S-M, respective (**b**–**d**) CD calibration curve.

UV–Vis spectroscopy revealed distinctive structural and surface features for the CDs-S sample. It exhibits a band at approximately 276 nm (see Figure 4), attributed to carbonyl (C=O) groups. These functional groups indicate the partial oxidation of sucrose during hydrothermal synthesis and play a key role in modulating the electronic structure and surface reactivity of the carbon quantum dots. Furthermore, C=C bonds are present within the sp^2^-hybridized carbon core, reflecting aromatic or graphitic subdomains that hinder electron delocalization, a typical characteristic of carbon quantum dots.

CDs-S-M exhibits an absorption band around 276 nm (see Figure 4), which is a resonant feature of surface plasmodons and the magnetite [Fe]_3_O_4_ nanoparticles. This band originates from collective electronic oscillations and reactions on the Fe^2+^/Fe^3+^-rich surface of magnetite and confirms the successful incorporation of the magnetic phase into the hybrid material, suggesting the presence of nanoscale magnetite domains with limited polydispersity.

The charge transfer processes between the carbonaceous and magnetic components, a property particularly relevant for biocatalytic applications mediated by redox processes and for the enhanced electron transfer dynamics observed in the CDs-S-M-Lac composite, are due to the formation of an electronically coupled hybrid system.

**Figure 5:**
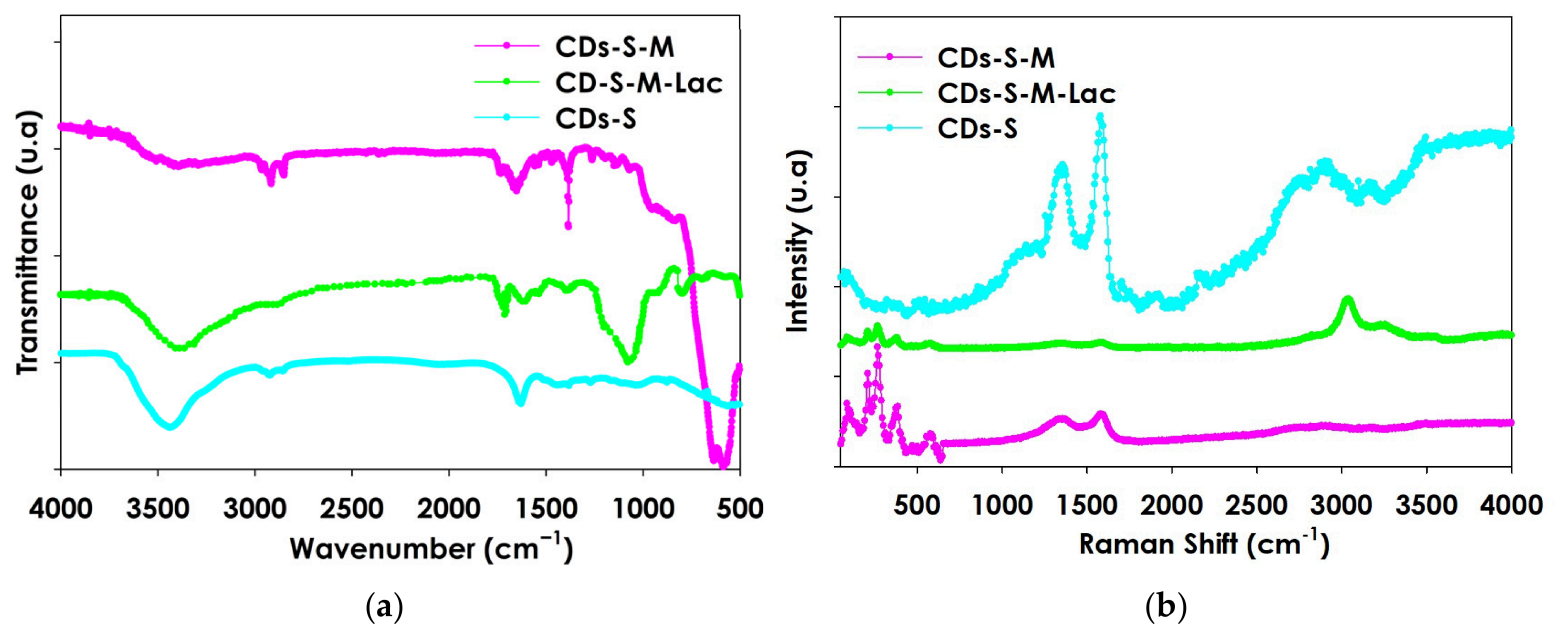
(**a**) FTIR spectra and (**b**) Raman spectra of CDs-S, CDs-S-M (composite with magnetite) and CDs-S-M-Lac (biocomposite).

Figure 5 shows the FTIR spectra of the CDs-S, CDs-S-M, and CDs-S-M-Lac samples, providing information on the chemical structures of the synthesized materials. The study is conducted in the 500–4000 cm^−1^ region. Different bands associated with functional groups are observed there, attributed to O–H stretching vibrations of hydroxyl groups. Likewise, the bands around 2850 cm^−1^ correspond to C–H stretching vibrations of aliphatic groups.

In the CDs-S sample, bands of 1625 cm^−1^ and 3410 cm^−1^ were found, respectively. The first is associated with alkene-type carbon groups, and the second with amine groups. Oxygenated functional groups are relevant because they are important for contaminant degradation. The absorption band of the CDs-S-M compound near 1378 cm^−1^ is associated with the C=C stretching vibrations (alkenes) characteristic of carbonaceous structures. Asymmetric stretching vibrations of carboxylate (COO^−^) groups were also observed. In the same sample, a band of 1651 cm^−1^ corresponding to carbonaceous groups was also obtained (see Figure 5a). The band around 2910 cm^−1^ indicates the presence of sulfonyl groups, which is essential in adsorption processes.

The presence of magnetite nanoparticles in the CDs-S-M-Lac compound is confirmed by the appearance of well-defined absorption bands at approximately 1075 cm^−1^ and 1702 cm^−1^. The first band is attributed to C–O and C–N stretching vibrations, while the band near 1702 cm^−1^ corresponds to the amide region. Finally, the absorption bands observed in the 500–790 cm^−1^ range are attributed to Fe–O stretching vibrations, thus confirming the presence of magnetite nanoparticles in the CDs-S-M and CDs-S-M-Lac compounds (see Figure 5).

Figure 5b presents the Raman spectra of the compounds. Both CDs-S and CDs-S-M exhibit the characteristic D (~1363 cm^−1^) and G (~1585 cm^−1^) bands, associated with sp^2^-hybridized carbon structures. The amplitude of these bands indicates the presence of nanocrystalline graphitic domains with a high density of sp^2^-hybridized defects. Furthermore, the marked increase in the spectral background in the 2500–3500 cm^−1^ region is attributed to the intense intrinsic photoluminescence of the carbon quantum dots, which hinders clear observation of the 2D band. The presence of the laccase enzyme in the biocomposite is confirmed by the appearance of characteristic Raman bands. In Figure 5b, a band at 3049 cm^−1^ is observed, corresponding to porous spaces, which underscores the importance of this research.

### 3.3. Atrazine Degradation Trials

To study atrazine degradation, the adsorption capacity of CDs-S and CDs-M materials was evaluated. Experiments were conducted at a controlled pH, as the experiment is sensitive to changes in the initial pH of the medium. Atrazine has a pKa value of approximately 1.7 and is predominantly found in its neutral molecular form. Although acidic conditions could favor atrazine–adsorbent interactions through partial protonation and electrostatic attraction, these conditions can lead to enzyme denaturation in the biocomposite system. For this reason, a solution pH of 5.0 was selected, representing a compromise between adsorption efficiency and enzyme stability, and widely reported as an optimum pH for laccase activity. It is important to note that the optimum pH is crucial for laccase activity.

**Figure 6:**
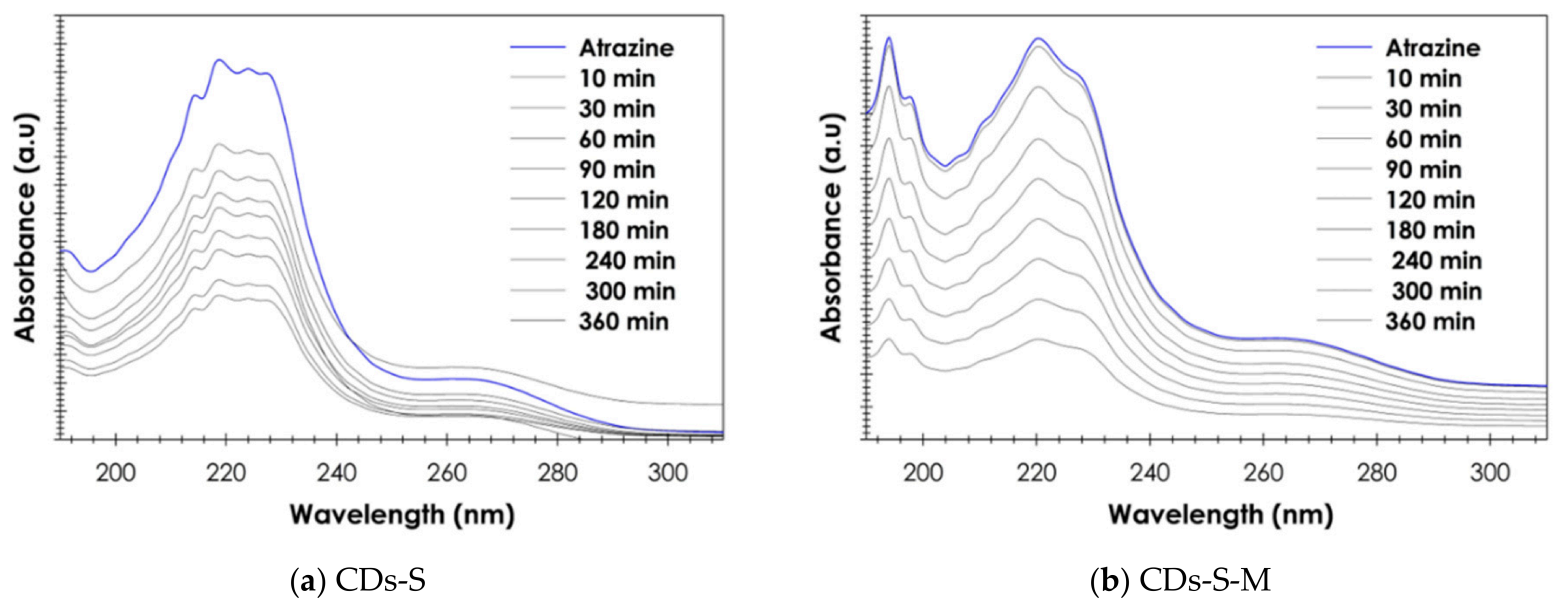
(**a**) Spectra of atrazine solution/CDs-S. (**b**) Spectra of atrazine solution/CDs-S-M.

Figure 6a shows that the initial concentration of atrazine ranged from 10 to 35 mg × L^−1^ during a contact time of 360 min. Figure 6b, corresponding to the CDs-S-M case, highlights the importance of using this type of adsorbent (especially magnetic ones) in the treatment of contaminated wastewater. This has generated considerable interest, since magnetic nanoparticles possess surface characteristics that contribute to the adsorption process, thus allowing for rapid and efficient separation of the contaminant from the medium. This is closely related to the Lambert–Beer law, which states that light absorption is proportional to the concentration of the substance.

**Figure 7:**
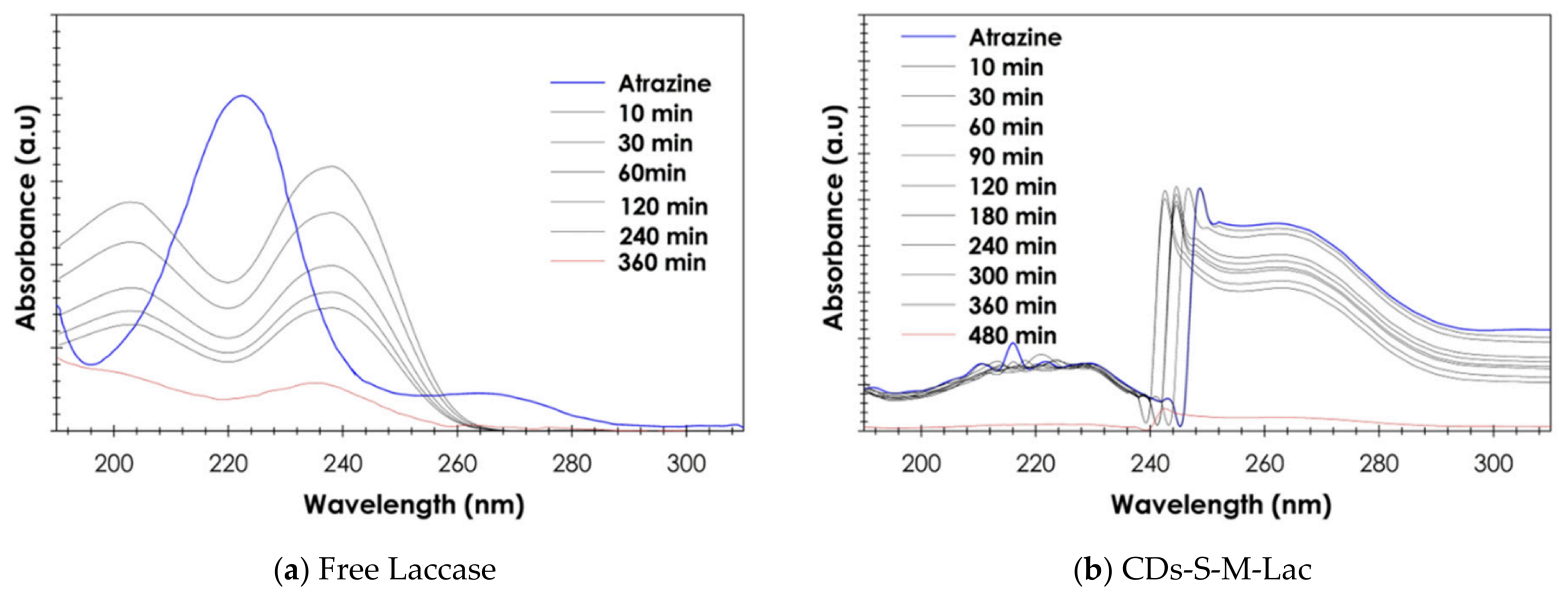
(**a**) Free Lacasse and (**b**) CDs-S-M-Lac.

Atrazine exhibits a characteristic UV absorption maximum around 222 nm (see Figure 7a), attributable to π → π* electronic transitions of the aromatic/heteroaromatic s-triazine system. When the molecule undergoes transformation processes (e.g., dealkylation, hydroxylation, or other routes that modify the electronic environment of the ring), absorption bands may appear or intensify in the ~235–240 nm region, which are typical of other s-triazines or derivatives with different substitution patterns (see Figure 7b). Therefore, the simultaneous decrease in the band at 220–222 nm and the broadening or growth of the signal towards ~240 nm are consistent with the formation of a mixture of reaction products and intermediates, rather than a simple dilution effect.

**Figure 8:**
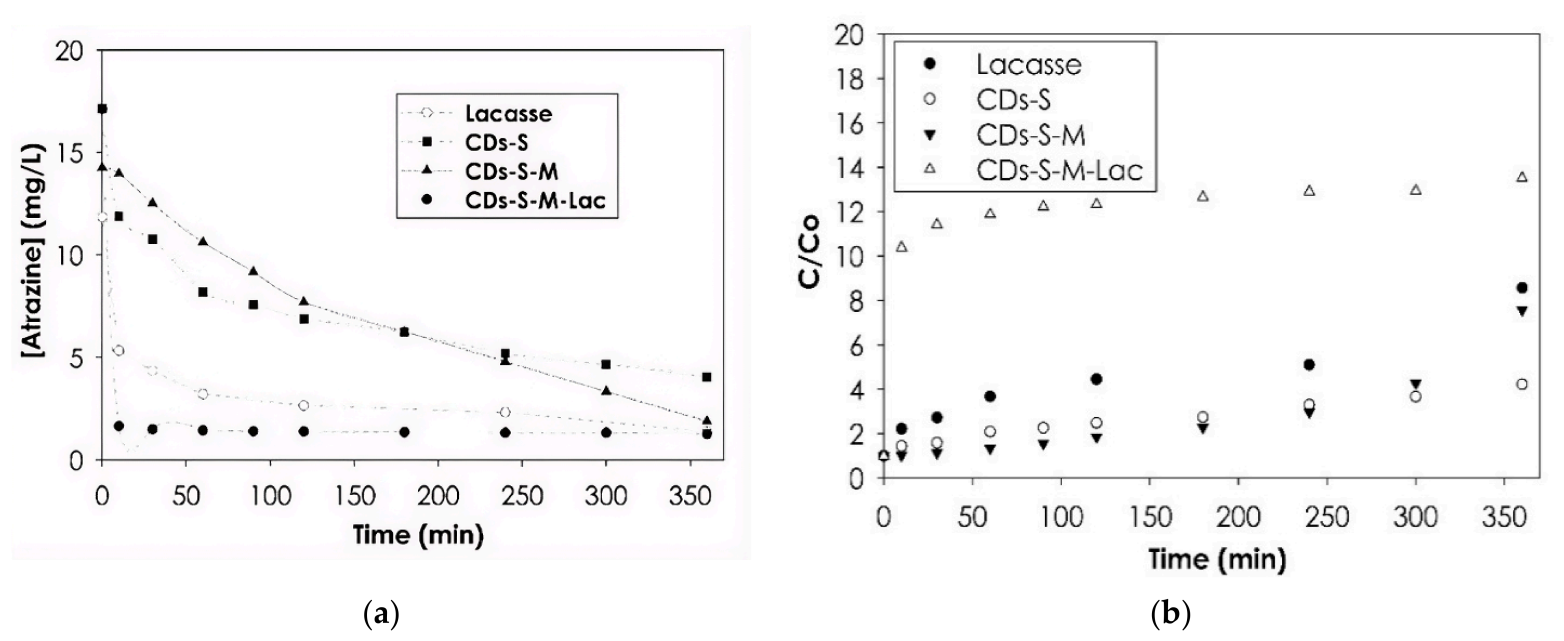
Representation of time versus change in atrazine concentration.

Looking at Figure 8, it can be observed that the atrazine concentration changes over time for each compound. The CDs-S-M-Lac sample demonstrates high degradation efficiency, as shown in Figure 6. Initially, the atrazine concentration is close to 17 mg/L, but it rapidly decreases by approximately 2 mg/L and remains stable until, after 360 min, it reaches 1.2 mg/L, being almost completely degraded. This contrasts with the other samples, which are less efficient, demonstrating the effectiveness of laccase in the adsorption process. However, CDs-S showed the lowest efficiency. Starting from an initial atrazine concentration of 17 mg/L, after 360 min, the concentration drops to 4 mg/L, having degraded 13 mg/L of atrazine. Atrazine degradation was evaluated using CDs-S, CDs-S-M, free laccase, and the hybrid compound CDs-S-M-Lac. Figure 8b represents the concentration of the sample over time, which complements Figure 8a since it is from there that the degradation of the contaminant is evaluated.

**Figure 9:**
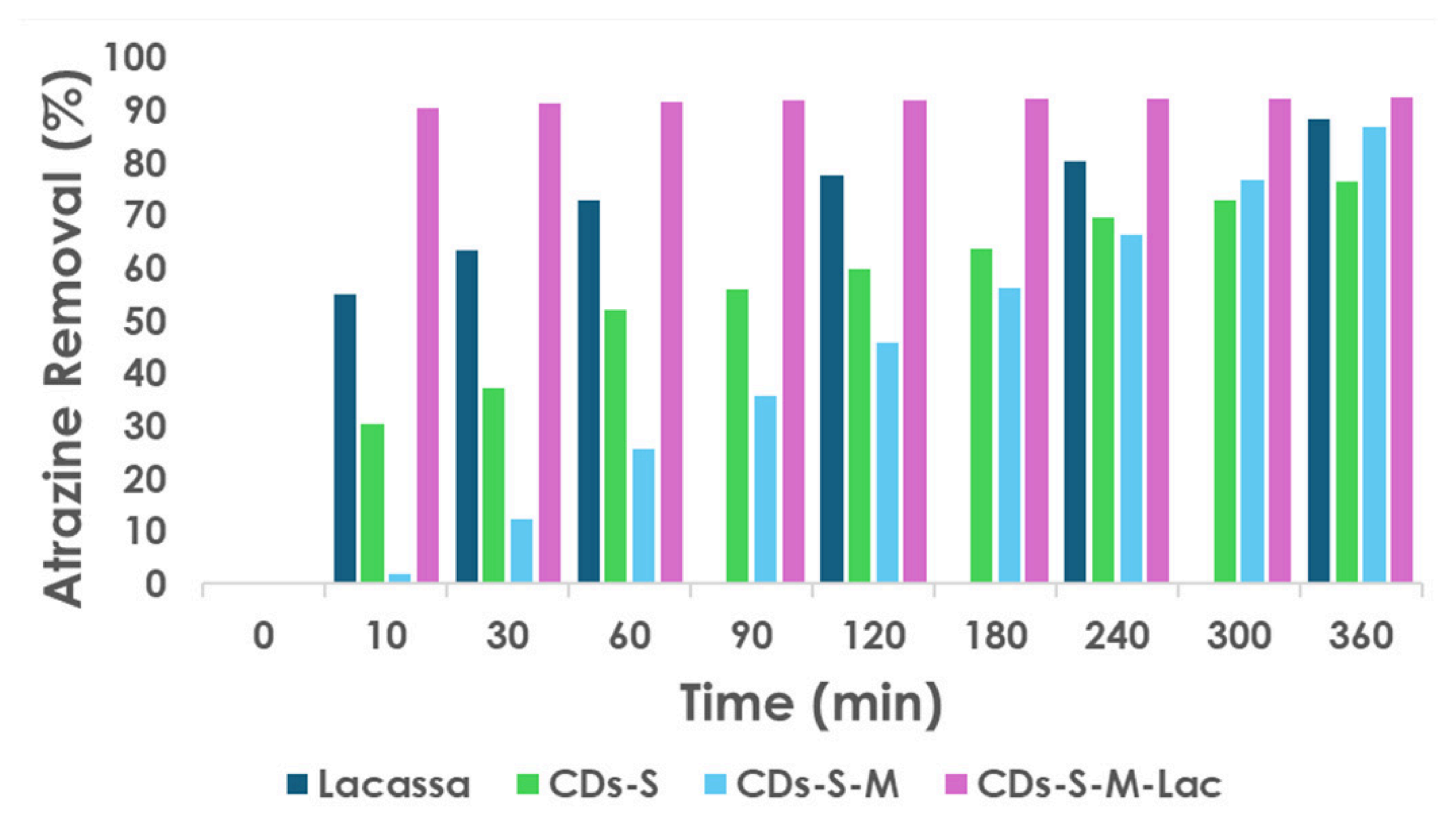
Compounds versus atrazine concentration variation.

Figure 9 shows that the CDs-S-M-Lac solid exhibits a higher atrazine degradation capacity. Importantly, this capacity remains stable and increases over time, as indicated in the bar graph. This suggests that laccase does not degrade. As a redox enzyme, it effectively oxidizes the contaminant without any change over time, unlike the other compounds. In contrast, the degradation capacity of the other samples varies over time, possibly due to intermolecular forces during the degradation process, but also due to pH sensitivity. The CDs-S-M sample is noteworthy because its atrazine degradation capacity is not significant in short periods, but over 360 min, it increases significantly, becoming equal to that of laccase alone. Therefore, the CDs-S-M-Lac material is effective in short periods.

## 4. Discussion

Carbon quantum dots (CDs) are carbon nanoparticles smaller than 10 nm with a wide range of applications. This research presents their ability to degrade atrazine, preparing a series of compounds, including CDs-S-M-Lac, which is the most effective atrazine degrader at a given time. This is attributed to its redox properties, which encapsulate and degrade the contaminant [15]. Furthermore, it is important to note that this is an environmentally friendly and novel method in the field of material’s engineering.

Carbon dots generate a nanostructured microenvironment around the laccase that performs multiple functions simultaneously. First, the CDs provide partial physical protection of the enzyme against denaturation and inhibition by reactive intermediates, helping to preserve the functional conformation of the active site. Second, the presence of surface electronic states and oxygenated and nitrogenous functional groups on the CDs can facilitate electron transfer processes at the nanoscale, favoring the oxidative activation of the substrate. Finally, nanozyme-like behavior cannot be ruled out, in which the CDs participate directly in parallel or synergistic redox reactions. This nanoshielding effect is also reflected in the temporal evolution of the UV–Vis spectra, as the progressive decrease in signals associated with intermediates suggests that the system prevents the accumulation of partially oxidized species, which are usually associated with enzyme inactivation processes [16].

It is important to compare this with other research, such as that presented by Gao et al., entitled “Laccase Immobilized On Arginine-Functionalized Boron Nitride Nanosheets Improves Atrazine Degradation,” which aimed to eliminate atrazine using laccase immobilized on boron nitride nanosheets. Their study achieved a reduction in atrazine concentration from 10 mg/L to 6.2 mg/L in 24 h [17]. Considering Figure 8, which shows the stability of contaminant degradation, the material presented in this research would eliminate 17 mg/L of atrazine, an amount even higher than that achieved by the aforementioned authors, who obtained a degradation of approximately 3.8 mg/L of atrazine, highlighting the importance of this research.

The Sustainable Development Goals are important to the theme of this research, as they highlight the importance not only of new materials but also of the sustainability of the process. Examples of this are the following goals [18]:

SDG 9—Industry, Innovation and Infrastructure: Quantum dots are innovative new materials that by degradate contaminants, representing an innovation.

SDG 14—Life below Water: Water pollution and its sources are a significant factor in the development of innovative solutions in both production and research.

SDG 17—Partnerships for the Goals: This involves forming partnerships so that these materials do not remain merely theoretical but can also be scaled up to a high-production stage. Even though they are new ideas, they need to be highlighted.

## 5. Conclusions

Atrazine degradation is achieved at 90% in any of the proposed timeframes for the CDs-S-M-Lac sample. The drastic reduction in concentration from 17 mg/L to 2 mg/L is novel and innovative, as other trials have not achieved this. This results in an efficient degradation of the contaminant, which can contribute to sustainability, since triazine is one of the world’s most potent pollutants. It is widely used as a broad-spectrum herbicide in agriculture to control weeds in crops such as sorghum and corn, but its prolonged use has contaminated water sources. Therefore, this research achieves up to 90% degradation using a CDs-S-M-Lac system.

Carbon quantum dots (CQDs) are promising nanomaterials for environmental remediation, notable for their low toxicity, high solubility, and large surface area. They are used for the degradation of heavy metals and, in this case, were proved to be relevant for atrazine degradation because their intermolecular bonds and interactions were effective in its elimination. However, further exploration and improvement in this research are needed because it is a relatively new field.

## Figures and Tables

**Table 1:** Preparation method of the different carbon dots.

Carbon Dots	Preparation Method
CDs-S	Carbon dots (CDs-S) were synthesized using a hydrothermal reaction method from sucrose and urea. A precursor solution was prepared by dissolving sucrose and urea in a 1.3:60 molar ratio in 40 mL of distilled water (the concentration of urea is 1.5 M and the concentration of sucrose is 0.0325 M), which was vigorously stirred for 30 min using a magnetic stirrer. Then, 0.5 g of PEG 3350 reagent was added, and stirring continued until complete dissolution. The resulting solution was transferred to a 100 mL stainless steel autoclave reactor with a Teflon lining and heated at 160 °C for 12 h. After natural cooling to room temperature, a brown CDs-S suspension was obtained, which was purified using a 3 kDa molecular weight cut filter. The resulting CDs-S solution was stored at 10 °C in a refrigerator.
CDs-S-M	These carbon dots were synthesized by adding FeCl_3_ · 6H_2_O to a precursor solution in a sucrose:urea:Fe^2+^ molar ratio of 1.3:60:6, dissolved in 40 mL of water (the concentration of sucrose is 0.0325 M, urea is 1.5 M and the concentration of Fe^2+^ is 0.015 M), and then vigorously stirred for 30 min. Subsequently, 0.5 g of PEG 3350 was added and stirred until completely dissolved. The solution was transferred to a 100 mL Teflon-lined stainless steel autoclave reactor and subjected to hydrothermal treatment at 160 °C for 12 h. After natural cooling to room temperature, a dark brown suspension of CDs-M was obtained. The particles were purified by magnetic separation for 10 min using a 0.20 T magnet, discarding the supernatant under the magnetic field. The purified suspension was stored at 10 °C for later use.
CDs-S-M-Lac	Laccase powder (10 mg) was dissolved in 20 mL of Sörensen phosphate buffer to maintain solution stability (PB: 30 mM NaH_2_PO_4_, 40 mM Na_2_HPO_4_, pH = 6.0) and stirred for 24 h at 4 °C. To remove the insoluble fraction, the solution was centrifuged for 20 min at 2000 rpm. Subsequently, 0.5 mL of the resulting laccase solution was mixed with 1 mL of CDs-S-M (0.75 mg mL^−1^) and stirred at 25 °C for 24 h, yielding the expected biocomposite CDs-S-M-Lac.

## Data Availability

The original contributions presented in this study are included in the article. Further inquiries can be directed to the corresponding author.

## Abbreviations

The following abbreviations are used in this manuscript:

Lac	Lacasse
M	Magnetite
S	Sucrose
CDs	Carbon Dots
